# Endemic bacteriophages: a cautionary tale for evaluation of bacteriophage therapy and other interventions for infection control in animals

**DOI:** 10.1186/1743-422X-9-207

**Published:** 2012-09-17

**Authors:** Andrew M Kropinski, Erika J Lingohr, Dianne M Moyles, Shivani Ojha, Amanda Mazzocco, Yi-Min She, Susan J Bach, Erica A Rozema, Kim Stanford, Tim A McAllister, Roger P Johnson

**Affiliations:** 1 Public Health Agency of Canada, Laboratory for Foodborne Zoonoses, 110 Stone Road West, Guelph, ON, N1G 3 W4, Canada; 2 Department of Molecular & Cellular Biology, University of Guelph, Guelph, ON, N1G 2 W1, Canada; 3 Centre for Vaccine Evaluation, Biologics and Genetic Therapies Directorate, Health Canada, Ottawa, ON, K1A 0 K9, Canada; 4 Agriculture and Agri-Food Canada, Pacific Agri-Food Research Centre, Summerland, BC, V0H 1Z0, Canada; 5 Department of Agricultural, Food and Nutritional Science, University of Alberta, Edmonton, AB, T6G 2P5, Canada; 6 Alberta Agriculture and Rural Development, Agriculture Centre, Lethbridge, AB, T1J 4 V6, Canada; 7 Agriculture and Agri-Food Canada, Lethbridge Research Centre, Lethbridge, AB, T1J 4B1, Canada

**Keywords:** *Escherichia coli* O157:H7, VTEC, Phage therapy, Phage ecology, Genome, Proteome, Bioinformatics, Morphology, Electron microscopy

## Abstract

**Background:**

One of the most effective targets for control of zoonotic foodborne pathogens in the farm to fork continuum is their elimination in food animals destined for market. Phage therapy for *Escherichia coli* O157:H7 in ruminants, the main animal reservoir of this pathogen, is a popular research topic. Since phages active against this pathogen may be endemic in host animals and their environment, they may emerge during trials of phage therapy or other interventions, rendering interpretation of trials problematic.

**Methods:**

During separate phage therapy trials, sheep and cattle inoculated with 10^9^ to 10^10^ CFU of *E. coli* O157:H7 soon began shedding phages dissimilar in plaque morphology to the administered therapeutic phages. None of the former was previously identified in the animals or in their environment. The dissimilar “rogue” phage was isolated and characterized by host range, ultrastructure, and genomic and proteomic analyses.

**Results:**

The “rogue” phage (Phage vB_EcoS_Rogue1) is distinctly different from the administered therapeutic *Myoviridae* phages, being a member of the *Siphoviridae* (head: 53 nm; striated tail: 152 x 8 nm). It has a 45.8 kb genome which is most closely related to coliphage JK06, a member of the “T1-like viruses” isolated in Israel. Detailed bioinformatic analysis reveals that the tail of these phages is related to the tail genes of coliphage lambda. The presence of “rogue” phages resulting from natural enrichments can pose problems in the interpretation of phage therapeutic studies. Similarly, evaluation of any interventions for foodborne or other bacterial pathogens in animals may be compromised unless tests for such phages are included to identify their presence and potential impact.

## Background

Foodborne microbial pathogens are a significant cause of morbidity and mortality globally, with a recent estimate placing the annual number of cases of foodborne disease at 11 million in Canada alone [1]. In an analysis accounting for under-reporting [2], estimates of the annual community rates of infections caused by the zoonotic foodborne pathogens *Salmonella*, *Campylobacter* and verotoxigenic *E. coli* in Canada are as high as 7, 19 and 3 per 1,000 population, respectively. While most individuals recover from these infections, longer-term health outcomes may include haemolytic uremic syndrome (HUS), chronic renal insufficiency, chronic arthritis, irritable bowel syndrome and Guillain-Barré syndrome.

The economic impact of these illnesses can be very high, with the annual cost to treat the short-term effects of acute gastrointestinal illness in Canada estimated to be about $1,089 CAD per case, with annual total costs exceeding $3.7 billion [3]. In the province of Ontario alone, the annual economic impact associated with human illness due to *E. coli* O157:H7 in ground beef has been estimated as $24.8 million [4]. Therefore, reducing human exposure to these pathogens would potentially have significant public health and economic impacts.

Given these impacts, substantial effort has been directed at controlling zoonotic foodborne pathogens early in the farm to fork continuum. Bacteriophages have been proposed, and used, as biocontrol agents in food animals and foods (reviewed in [5-13]. Recently, their potential as therapeutics convinced the United States Food and Drug Administration to allow the use of Intralytix’s anti-*Listeria monocytogenes* phage cocktail ListShield in meat products [14]. Similarly, LISTEX^TM^ (a phage preparation from MICREOS Food Safety (Netherlands)) was approved as GRAS (Generally Recognized As Safe) and is currently being used in the ready-to-eat food industry in North America. OmniLytics (Salt Lake City, UT) has obtained approval from the United States Department of Agriculture/Food Safety & Inspection Service for a hide-washing solution containing phage active against *E. coli* O157:H7; and more recently for *Salmonella*.

The process of bacteriophage therapy for pathogen control is dependent upon the selection of broad-range lytic bacteriophages with rapid adsorptive properties and strong cell killing activity. Furthermore, the phages must be capable of being mass produced, stable during preparation and storage, and shown to be safe and effective upon application. Like many other interventions such as vaccines, probiotics and other antimicrobials, bacteriophage therapy for control of foodborne zoonotic pathogens is often evaluated in experimentally or naturally infected animals. During such evaluations it is imperative to ensure that any factors that may confound assessment of the true effectiveness of the tested intervention are carefully monitored. It is now well known that animals and their environments are natural niches of bacteriophages. For example, *E. coli* O157:H7 bacteriophages are ubiquitous in feedlot cattle and their environment, and at very high prevalences when identified by phage enrichment using rather simple screening tests [15-17]. Consequently, it has been recommended that before *in vivo* pathogen eradication studies using phage or any other regime, test animals should be enrichment screened for phages to avoid erroneous results [15]. In 2009, our group first described the appearance of lytic phages distinct from therapeutic phages administered to steers preinoculated with 5 x 10^10^ CFU of a mixture of *E. coli* O157:H7 strains [18]. These “rogue phages” produced larger plaques than the therapeutic phages and were morphologically distinct. Also, they appeared to be endemic, since the same phages had been isolated from *E. coli* O157:H7-inoculated sheep in a preliminary phage therapy trial on the same facility four years earlier. In the current manuscript we describe the physiological, genetic and proteomic characteristics of one of these viruses, and comment on the potential impact of endemic phages on evaluation of phage therapy and other interventions for control of bacterial infections.

## Results

### Isolation, plaque morphology and host-range of vB_EcoS_Rogue

**Figure 1:**
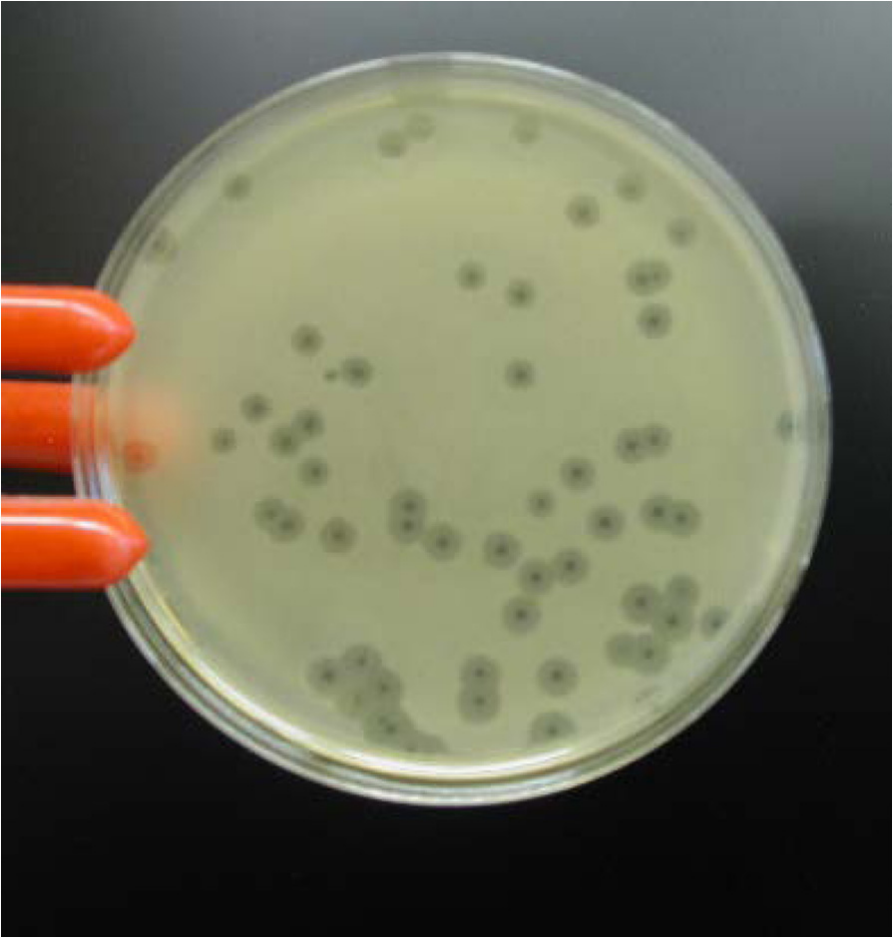
**Photomicrograph of plaques produced by phage Rogue1 on lawns of *E. coli *O157:H7 R508N.**

Three isolates of the rogue phage were successfully propagated and enumerated on strain R508N using standard protocols [19]. Their plaques averaged 4 mm in diameter wide opalescent zones with around 1 mm centres of clear lysis (Figure 1), closely resembling the rogue phage identified in cattle [18] as compared to the 1-2 mm clear lytic plaques of the administered phages. In host-range studies, the three rogue phage isolates possessed essentially identical host ranges, being virulent for most tested strains except for the two non-O157 strains and one *E. coli* O157:H7 PT1 strain (Table 1). All three gave an identically sized amplicon upon PCR analysis using primer pairs directed to the putative tail fibre gene (data not shown).

**Table 1:** ***E. coli *strains used to assess the virulence of the three isolates of the rogue phage as determined by the microplate virulence assay**

**Strain No.**	**Serotype**	***E. coli *O157 Phage Type**	**Strain description**	**Minimum Phage MOI**^**1**^		
				**LRCSh151d1**	**LRCSh158d1**	**LRCSh166d1**
EC19990293	O157:H7	PT21	Phage type reference strain	0.00001	0.000001	0.000001
EC19990295	O157:H7	PT4	Phage type reference strain	0.00001	0.00001	0.000001
EC19990296	O157:H7	PT23	Phage type reference strain	0.000001	0.000001	0.00001
EC19990298	O157:H7	PT14	Phage type reference strain	0.00001	0.000001	0.000001
EC19990299	O157:H7	PT14	Phage type reference strain	0.01	0.00001	0.001
EC19990300	O157:H7	PT2	Phage type reference strain	0.0001	0.0001	0.00001
EC20010992	O7:H21	NA^2^	Non-pathogenic *E. coli*	>10	>10	>10
EC19990779	O173:HUN	NA	Non-pathogenic *E. coli*	>10	>10	>10
EC20030480	O157:H7	PT4	Challenge strain LRC.E318N	0.000001	0.000001	0.000001
EC20030479	O157:H7	PT1	Challenge strain LRC.E319	>10	>10	>10
EC20030478	O157:NM	PT87	Challenge Strain LRC.E32511N	10	10	10
EC20030477	O157:H7	PT87	Challenge Strain LRC.H4420nal	0.000001	0.00001	0.000001
EC20030481	O157:H7	PT14	Challenge Strain LRC.R508N	0.00001	0.00001	0.000001
EC19940312	O157:H7	PT8	Challenge Strain LCDC.CO281–31nal	0.000001	0.0001	0.01

^1^ MOI: Multiplicity of Infection required for complete lysis of 1 CFU of the tested strains by the listed phages. Phages were identified as virulent for a bacterial strain when the MOI was ≤10. ^2^ NA: not applicable. Description: Table (Microsoft Word).

Using the convention established by Kropinski et. al. [20], and electron microscopic observations, this virus was formally called vB_EcoS_Rogue1, but will subsequently be referred to as Rogue1 in this manuscript.

### Electron microscopy

**Figure 2:**
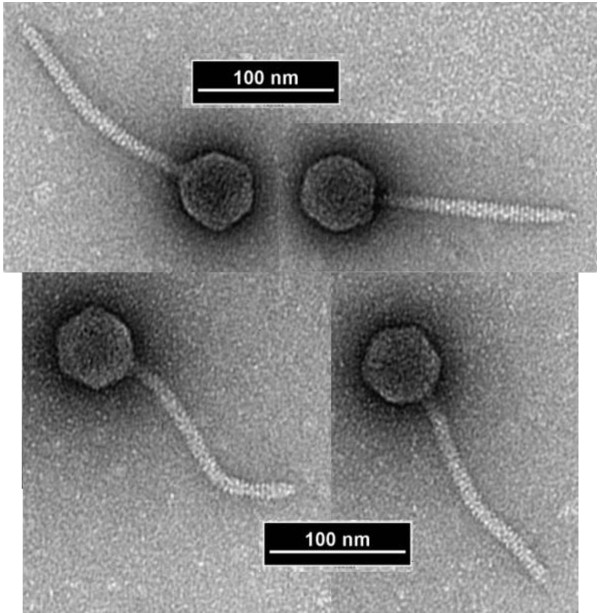
**Four representative electron micrographs of Rogue1 negatively stained with 1% uranyl acetate showing that they belong to the family *Siphoviridae *with morphology reminiscent of coliphages λ or T1.**

Negatively-stained preparations of phage Rogue1 displayed typical siphovirus morphology with long, relatively rigid (152 x 8 nm) noncontractile tails (Figure 2). Tail striations have a periodicity of 3.5 nm and terminated in short spikes. Fixation with ammonium molybdate resulted in tails which curled up (data not shown). The heads measured 53 nm in diameter and displayed a constriction at the neck. These dimensions again closely resemble that of the cattle rogue phage [18]. By comparison, the head of coliphage T1 is about 60 nm in diameter and the tail is 8 by 151 nm [21].

### DNA sequencing and genome characteristics

The genome size of Rogue1 was estimated to be 44.3 kb by pulsed-field gel electrophoresis. Pyrosequencing has become a routine and inexpensive way of sequencing bacteriophage genomes. The sequence of phage Rogue1 resulted in two contigs of 12.8 (117 fold coverage) and 33.6 kb (99 fold coverage), respectively. Using custom PCR primers we amplified and sequenced the missing section resulting in collapse of the sequence data into a single contig. In a similar manner, two 454 sequencing induced frameshifts were corrected.

**Figure 3:**
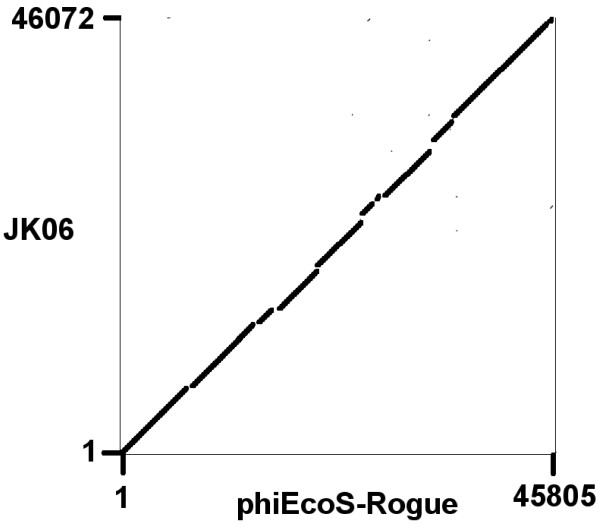
Dotplot comparison of the genomes of Rogue1 and JK06 showing essentially collinear genome sequences.

It was immediately recognized that the sequence of Rogue1 was closely related to that of JK06 (NC_007291) in that they shared genome size, mol%G + C and a single arginyl-tRNA - Rogue1 (45.8 kb, 44.2 mol%G + C) and JK06 (46.1 kb, 44.0 mol%G + C). For comparative purposes, the completed sequence of Rogue1 was reordered so that it began with the same nucleotide sequence “acgcgtatatcaaat-agcac” as that of coliphage JK06. However, direct comparisons between Rogue1 and JK06 sequences were complicated due to numerous errors contained in the JK06 sequence and its annotation. From our perspective, JK06 contains 19 genes which should be deleted, some of which are due to frameshifts. As a consequence of the frameshifts, five of the JK06 genes should therefore be reclassified as pseudogenes. A dotplot comparison of the two genomes presented in Figure 3 indicates they are collinear. Using bl2seq [22] at http://blast.ncbi.nlm.nih.gov/bl2seq/wblast2.cgi the alignment revealed nine segments of >1 kb which showed 95-99% identity. Based upon an analysis of 84.1% of the Rogue1 genome length, the sequences are approximately 96.9% identical.

Bioinformatic analysis revealed the presence of 74 protein-encoding genes and an arginyl-tRNA (genome position 3387-3462) recognizing the rare codon AGG [23]. The majority (62, 83.8%) of the proteins exhibit homology to other T1-like phages, 26 of which have defined function in DNA replication, morphogenesis, genome packaging and lysis (see Additional file 1, Table S1).

### Proteomics

**Figure 4:**
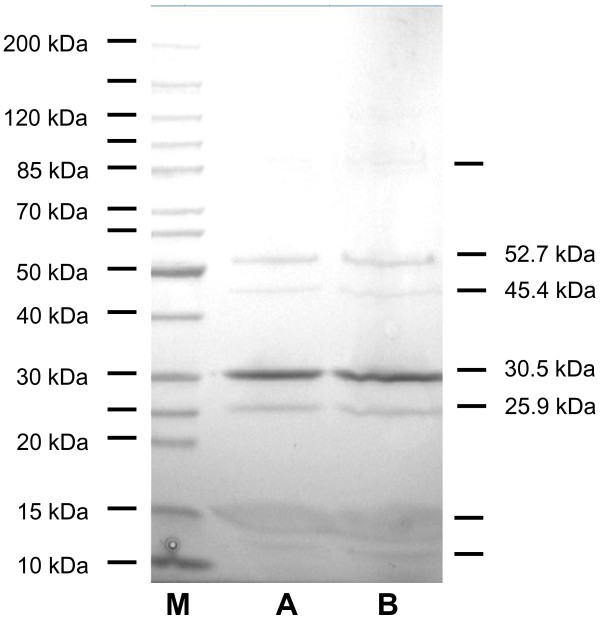
**SDS-PAGE of phage Rogue1 structural proteins.** Lanes A and B contain samples of the virus while the lane labelled M has the Fermentas PAGERuler.

The proteins of purified phage particles were resolved by SDS-PAGE, revealing approximately six bands (Figure 4). Proteomic analysis of the three indicated phage bands (52.7 kDa, 30.5 kDa & 25.9 kDa) by MALDI QqTOF mass spectrometry identified proteins that exactly matched the predicted sequences of the proteins specified by genes *21*, *22*, and *29* ( Additional file 2, Figure S1) revealing a high sequence coverage of 59.5%, 83.5% and 53.7% in the tryptic digests, respectively.

The gene for the major head protein specifies a 39.8 kDa product, yet SDS-PAGE (Figure 4) revealed that this protein migrated with an estimated size of 30.5 kDa; and mass spectrometry ( Additional file 2, Figure S1; Additional file 3, Table S2) did not reveal the N-terminal tryptic peptides. These results suggest that the major capsid protein is enzymatically removed during morphogenesis, perhaps through the action of the product of gene *18*.

Analysis of the 57.8 kDa protein (Gp21) revealed four repeats of the pentamer GPVGP. A ClustalW alignment ( Additional file 4, Figure S2) with its homolog phage JK06 protein YP_277466.1, which is incorrectly described as “hypothetical DNA polymerase I,” reveals that these repeats are found at the borders and internally to a region which is not found in JK06. This finding suggested the presence of an intein, however using data on intein structure from InBase (The Intein Database) [24] at http://www.neb.com/neb/inteins.html showed none of the residues usually associated with intein boundaries was present.

## Discussion

This example of ruminants acting as incubators for the enrichment of pathogen-specific bacteriophages resembles the studies from Bangladesh in which humans act as incubators for *Vibrio cholerae*-specific phages [25,26]. These viruses presumably occur in the environment at subdetectable levels and are enriched when a sufficiently high host inoculum is provided. Similar situations involving viruses active on phytopathogens where the ecosystems adjacent to infected plants were reported to have a higher concentration of specific phages than soil or water from the vicinity of uninfected plants [27]. The potential presence of enriched endemic bacteriophages can make interpretation of the efficacy of a phage cocktail extremely problematic. In our case *E. coli* O157:H7-inoculated sheep in a phage therapy trial were found to shed a morphologically distinct phage which we have characterized genomically and proteomically in this manuscript. The emergence of an identical phage four years later on the same facility [18] strongly suggests it is endemic. Other endemic phages have been isolated and characterized by Niu et al. [28,29] and Raya et al [30]. These phages would not have been detected without testing for phage shedding, a practice not typically conducted in evaluation of other interventions. Consequently, the potential impact of endemic phages on the outcomes of any therapeutic trials would not be identified, an observation that supports the cautionary recommendations for inclusion of phage testing in any *in vivo* intervention studies (15).

In the Ninth Report of the International Committee on Taxonomy of Viruses, the *Siphoviridae* are assigned to eight bacteria-specific genera of viruses (Lambda-, PhiC31-, c2-, L5-, N15-, SPbeta-, T1-, and T5-like viruses) and one archaeal-specific genus represented by *Methanobacterium* phage ΨM1 [31]. On the basis of DNA and protein comparisons there is no doubt that coliphages JK06 and vB_EcoS_Rogue1 belong to the “T1-like viruses” genus along with coliphages Rtp [32], TLS [21], and phiEB49 (JF770475), *Cronobacter* phage ESP2949-1 [33], *Enterobacter aerogenes* F20 (JN672684), *Klebsiella* phage KP36 (Z. Drulis-Kawa, personal communication), and *Shigella* phage Shfl1 (NC_015456). Whole genome proteomic comparisons using CoreGenes [34] revealed that T1 shares 42 proteins in common with Rogue1 (53.8% similarity) with the homologs generally evenly distributed except for the first 18 and last six T1 genes for which no homologs exist in Rogue1. Unfortunately, an accurate comparison of the proteomes of JK06 and Rogue1 cannot be made due to the errors found in the former sequence but their close similarity in nucleotide sequence suggests that they are variants of the same species. Here we have two phages which were isolated in Israel (JK06) and Canada (vB_EcoS_Rogue1) which are remarkably similar at the DNA level. A number of other presumably related phages have recently been characterized microscopically and on the basis of restriction digestion profiles [29]. These observations indicate that related phages are distributed globally as has been shown previously with *Pseudomonas* phage PB1 [35] and φKMV [36].

BLAST analysis on the proteins found in the tail region of phage Rogue1 resulted in the discovery of homologs of coliphage lambda gpH, gpL and gpI. Homology detection & structure prediction by HMM-HMM comparison (HHpred) analysis [37,38] revealed that Rogue1 genes *26*, *28*, and *29* specified proteins were structurally related to lambda gpFII, gpU, and gpV. Moreover, the arrangement of the lambda tail morphogenesis cluster is identical to that of our phage with the exception of the presence of gene *34*. Based upon synteny, we hypothesize that Rogue1 genes *27*, *30*, *31*, *33* and *36* specify analogues of lambda gpZ, G, T, M and K. While gene *30* does not possess the classical lambda frameshifting site (GGGAAAG) it does possess a downstream pseudoknot structure and therefore during translation may form a G-T-type fusion protein with gp31. Furthermore, part of Phyre^2^[39] analysis of the potential 3D structure of gp33 and accompanying PSI-BLAST search indicated one of the hits was to UniRef50 (UniProt NREF (UniProt Reference Clusters) database) [40,41] P03737 (E-value: 3e-23). This protein is identified as lambda minor tail protein M. While there is no proteomic evidence for the presence of gpI, L and M in the tail of coliphage lambda [42] these proteins are indeed involved in the assembly of the tail. These results collectively provide a genetic basis for the morphological quasi-identity of lambda and T1 that was noted in 1974 by Christensen [43]. Furthermore, in uranyl acetate, lambda and T1 look absolutely identical; while in phosphotungstate, the lambda tail is rather rigid and that of T1 is extremely flexible (Ackermann, unpublished results). The difference is clearly in the tail shaft protein. Lastly, we were able to successfully model the structure of 99 residues (40%) of Rogue1 gp36 ( Additional file 5, Figure S3**.**) with 100.0% confidence against a single highest scoring template (RCSB PDB number 2EVR). The latter is putative gamma-D-glutamyl-l-diamino acid endopeptidase [44]. If this is part of the distal end of the phage tail it may play a role in degradation of the peptidoglycan sacculus during injection as noted with other phage tail proteins [45-47].

## Materials and methods

### Experimental Animals and Treatments

Five Canadian Arcott rams (50.0 + 3.0 kg) were housed in a separate room in a contained facility equipped with its own feed bunk and water source. They were fed a pelleted barley-based total mixed diet once daily and were adapted to their environment for a period of seven days prior to the start of the trial. During this time feces of the five sheep were screened for *E. coli* O157:H7 and *E. coli* O157:H7 phages by established enrichment methods [18]. None was detected. On Day 0, the sheep were infected orally with 10^9^ CFU of *E. coli* O157:H7 R508N, a nalidixic acid-resistant phage type 14 strain, followed on Days 4 and 5 by ruminal inoculation with 10^10^ PFU of a cocktail of three lytic *E. coli* O157:H7 phages; rV5, wV8 and wV7. Fecal samples collected from all sheep daily for seven days then every second day to Day 22 were tested for *E. coli* O157:H7 and *E. coli* O157:H7 phages as described [18]. Care and use of the animals throughout the study were approved by the Animal Care Committee of the Lethbridge Research Centre of Agriculture and Agri-Food Canada under Animal Use Protocol 0344, and in accordance with guidelines established by the Canadian Council on Animal Care (1993).

### Isolation of Rogue phage

Feces from three of the five sheep tested on Day 5 contained phages forming plaques with a 1 mm zone of clear lysis and a large opalescent halo, distinctly different from the 1-2 mm plaques of the administered phages that were also present. These “rogue” phages were subsequently shed by two or more sheep at each sampling between Days 5 and 18 at levels of ~10^1^ to 10^4^ PFU/g of feces, together with the small plaque-forming administered phages at levels of ~10^1^ to 10^7^ PFU/g. One of the rogue phages from each of the three sheep on the first day of shedding was isolated on R508N and were named LRCSh151d1(R508N), LRCSh158d1(R508N), and LRCSh166d1(R508N), respectively. They were subsequently propagated on strain R508N, plated on nutrient agar and their unique plaque morphology photographed.

### Host-range study

After propagation, the host-ranges of the phages were assessed using a microwell virulence assay [28] by incubating 10^8^ bacterial cells with 10-fold serial dilutions (10^9^ to 10^2^) of each bacteriophage in Tryptic Soy Broth (TSB: BD Canada, Mississauga ON, Canada). Following a five hour incubation at 37°C, the minimum number of phages required to completely lyse 1 CFU of the strain was determined [28]. The tested strains included reference strains of *E. coli* O157:H7 phage types common in Canada, two potential non-pathogenic *E. coli* host strains (O7:H21 and O173:HUN) and six nalidixic acid resistant strains of *E. coli* O157:H7 planned for used for multiple strain challenge studies (Table 1). Phages were identified as virulent for a bacterial strain when the MOI was equal to or less than 10.

### Electron microscopy

A purified sample of phage was diluted 1:1 with distilled water and 10 μL were immediately transferred to a formvar/carbon coated copper grid. Excess sample was wicked off with filter paper and the grid was stained for 20 sec. with 1% uranyl acetate (aq). Excess stain was removed using filter paper and the grid was allowed to dry before putting it into the microscope. The photographs were taken on the LEO912AB transmission EM at 100 kV using a Cantega 2 K camera.

### Purification, DNA isolation and DNA sequencing

The phage was batch cultivated in 2.0 L of Trypticase soy broth containing 10 mM MgSO_4_ for 18 h at 37°C with shaking at 120 rpm. The resulting lysate was clarified by centrifugation (6,000 x *g*), treated with DNase 1 and RNase A (Sigma Aldrich Canada Ltd., Oakville, ON) and concentrated by 10% w/v polyethylene glycol precipitation [48]. The phage was purified through two rounds of CsCl equilibrium gradient centrifugation as described in [49], followed by dialysis and spectrophotometric quantitation.

DNA was isolated from LRCSh151d1(R508N) using the standard phenol-chloroform extraction protocol [49]. The DNA was subjected to pyrosequencing at the McGill University and Genome Québec Innovation Centre (Montreal, QC, Canada) resulting in two contigs. The 90 bp gap was closed by PCR amplification using custom primers and ABI sequencing at the University of Guelph Laboratory Services (Guelph, ON, Canada). Two genome regions suggesting frameshifts were also investigated by PCR and amplicon sequencing.

In addition the genome size was estimated by pulsed field gel electrophoresis [50].

### Annotation and comparative genomics

Protein-encoding genes for phage vB_EcoS_Rogue1 were identified using Kodon (Applied Maths, Austin, TX), while tRNAscan-SE [51] and ARAGORN [52] were used to identify tRNA genes. All proteins were checked for homologs against the nonredundant database at NCBI using Batch-BLAST ( http://greengene.uml.edu/programs/ NCBI_Blast.html). Protein motifs were examined using Pfam [53], TMHMM [54] and Phobius [55]. In the case of conserved hypothetical proteins they were analyzed using HHpred at http://toolkit.tuebingen.mpg.de/hhpred and for tertiary structure using Phyre^2^[39]. Rho-independent terminators were discovered using TransTerm [56] at http://nbc11.biologie.uni-kl.de/framed/left/menu/auto/right/clusterinfo2/www/. Promoters were located on the basis of homology to the consensus sequence (TTGACA(N15-17)TATAAT) of sigma70(RpoD)-dependent *E. coli* promoters using DNAMAN (Lynnon Corp., Pointe-Claire, QC, Canada).

For comparative purposes pairs of phage DNA sequences were aligned using Advanced Pipmaker [57] and MAUVE [58]; while the proteomes were compared using CoreGenes [34].

### PCR analysis of putative tail fibre genes

Two primers (JK-75-F, 5’-TAGGAA TGCCGGAAACTGTAGGAT and JK-75-R, 5’-CGTTTGCTGGCTTAATTCTTG TCT) based upon orf*75* of phage JK06 (NC_007291) were used with 1X PCR buffer, 400uM dNTPs, 3.5 mM MgCl_2_, 300nM primers, and 0.05U/ul Taq polymerase (Applied Biosystems, Branchburg, NJ, USA) under the standard to amplify a 509 bp product from the rogue phage genomes.

### GenBank accession number

The sequence of phage vB_EcoS_Rogue1 was exported from Kodon and subjected to a conversion using gbk2sqn ( http://lfz.corefacility.ca/gbk2sqn/) prior to submission to NCBI. The accession number for genome sequence of phage vB_EcoS_Rogue1 is JQ182736.

### Proteomics

CsCl-purified phage particles were subjected to SDS-PAGE [59] on precast 4-15% gradient TRIS acrylamide gels (BioRad) along with the PageRuler Unstained Protein Ladder (Fermentas, Burlington, ON, Canada). The gels were stained with SimplyBlue SafeStain (Invitrogen) and analyzed using BioNumerics software (Applied Maths). For more detailed proteomic analysis individual phage bands were excised from the gels and digested with trypsin as described previously [60]. In brief, the protein bands were first destained with 100 mM ammonium bicarbonate (NH_4_HCO_3_)/acetonitrile (ACN) (1:1, v/v) until colorless, then followed by reduction with 10 mM dithiothreitol (DTT) at 56°C and alkylation with 55 mM iodoacetamide in 100 mM NH_4_HCO_3_ solution. Subsequent digestion was performed using 10 ng of sequencing grade trypsin (Calbiochem, San Diego, CA) in 25 mM NH_4_HCO_3_ (pH 7.6) at 37°C overnight. The resulting peptide digests were extracted by successive sonication with 0.1% TFA and 0.1% TFA in 60% ACN, and dried in the Savant SpeedVac concentrator. After C18 Ziptip (Millipore) cleaning, the tryptic peptides were analyzed by matrix-assisted laser adsorption ionization (MALDI) mass spectrometry using 2,5-dihydroxybenoic acid matrix (100 mg/ml in 50% ACN). All mass spectrometry (MS) experiments were carried out on a Applied Biosystems/MDS Sciex QStar XL QqTOF mass spectrometer equipped with oMALDI II source and a nitrogen UV laser (337 nm). Following MS mapping, tandem MS/MS sequencing was routinely performed on the individual peptides at low-energy CID using argon as the collision gas. Protein identification was achieved by Mascot database search ( http://www.matrixscience.com) with the masses of either single MS peptide mass fingerprinting or MS/MS fragments based on the NCBI nonredundant database and the in-house *Escherichia coli* O157:H7 phage Rogue1 database. The search parameter settings allowed trypsin digestion for maximum 2 missed cleavage sites, and carbamidomethylation of cysteine as a fixed modification. Deamidation of asparagine and glutamine, oxidation of methionine, and loss of methionine at protein N-terminus were considered as variable modifications. Mass tolerances were set up to 0.1 Da for both MS and MS/MS fragment ions.

## Abbreviations

BLAST: **B**asic **L**ocal **A**lignment **S**earch **T**ool; CID: Collision-induced dissociation; CFU: Colony forming unit; ESI-MS/MS: electrospray ionization tandem mass spectrometry; Gp: Gene product; HHpred: Homology detection & structure prediction by HMM-HMM comparison; MOI: Multiplicity of Infection, ratio of infective phage particles to vulnerable hosts; NA: Not applicable; PCR: Polymerase chain reaction; PFGE: Pulsed field gel electrophoresis; PFU, Plaque Forming Unit: a measure of the number of viable viral particles; QqTOF: Quadrupole time-of-flight; SDS-PAGE: denaturing (sodium dodecyl sulfate) polyacrylamide gel electrophoresis; TMHMM: **T**rans**M**embrane prediction using **H**idden **M**arkov **M**odels.

## Competing interests

The authors have no competing interests to disclose.

## Authors’ contributions

EJL and AM characterized the rogue phage at LFZ, EJL performed the PFGE, SO prepared the phage DNA for sequencing, AMK performed DNA sequence analyses, Y-MS analyzed the proteome, DMM did the initial EM work with the sizes corroborated by H-WA. SB and TAM isolated and examined the first rogue phage from sheep (unpublished) and with ER and KS isolated the same phage from a cattle study four years later with therapeutic phages prepared by RJ and AM. AMK and RPJ wrote the manuscript with input from the other authors. All authors read and approved the final manuscript.

## Supplementary Material

Additional file 1 **Table S1.** Properties of the proteins encoded by phage Rogue1. Click here for file

Additional file 2 **Figure S1.** Mass spectral analysis of three Rogue1 structural proteins. Click here for file

Additional file 3 **Table S2.** Protein sequence identification of Rogue1 phage by MS/MS measurements of the peptides and database search. Click here for file

Additional file 4 **Figure S2.** ClustalW alignment of Rogue1 gp21 with its homolog phage JK06 protein YP_277466.1. The pentameric repeats are indicated in bold and green. Click here for file

Additional file 5 **Figure S3.** 3D structure of a portion of gp36 based upon template c2fg0B, determined using Phyre^2^[39]. Click here for file
