# Supplementary material for: Endemic bacteriophages: a cautionary tale for evaluation of bacteriophage therapy and other interventions for infection control in animals
Source: Virol J. 2012 Sep 17;9:207. doi: 10.1186/1743-422X-9-207 (PMC3496638; doi:10.1186/1743-422X-9-207)
Supplement: Additional file 1 — Table S1. Properties of the proteins encoded by phage Rogue1. [file 1743-422X-9-207-S1.doc]

Additional file 1: Table S1. Characteristics of the proteins encoded by phage Rogue1.

| **Gene Name** | **Coordinates** | **Strand** | **Length (nt)** | **Protein mass (Da)** | **pI** | **AA residues** | **Function** | **Homologs & motifs** | **BLAST E value** | **HHPred hit** | **Probability** |
| --- | --- | --- | --- | --- | --- | --- | --- | --- | --- | --- | --- |
| 1 | 66..260 | + | 195 | 8107 | 9.6 | 64 | hypothetical protein |  |  |  |  |
| 2 | 420..542 | + | 123 | 4625 | 7.8 | 40 | hypothetical protein |  |  |  |  |
| 3 | 561..1103 | + | 543 | 20793 | 6.5 | 180 | conserved hypothetical protein | YP_277487.1| hypothetical protein JK_47 [Enterobacteria phage JK06] | 2.00E-77 |  |  |
| 4 | 1103..1189 | + | 87 | 3434 | 9.2 | 28 | hypothetical protein |  |  |  |  |
| 5 | 1225..1500 | + | 276 | 10456 | 4.8 | 91 | conserved hypothetical protein | YP_277484.1| hypothetical protein JK_44 [Enterobacteria phage JK06] | 6.00E-31 |  |  |
| 6 | 1539..1634 | + | 96 | 3598 | 6.0 | 31 | conserved hypothetical protein | C-terminus AEI91271.1| gp71 [Escherichia phage phiEB49] | 6.00E-10 |  |  |
| 7 | 1619..1735 | + | 117 | 4551 | 3.5 | 38 | hypothetical protein |  |  |  |  |
| 8 | 1753..2052 | + | 300 | 11284 | 8.7 | 99 | conserved hypothetical protein; putative chaperone | YP_277483.1| hypothetical protein JK_43 [Enterobacteria phage JK06] | 5.00E-68 | 1we3_A CPN60(groel); chaperone | 89.6 |
| 9 | 2125..2253 | + | 129 | 5165 | 4.8 | 42 | conserved hypothetical protein | AEI91268.1| gp68 [Escherichia phage phiEB49] | 4.00E-18 |  |  |
| 10 | 2250..2543 | + | 294 | 11012 | 4.7 | 97 | conserved hypothetical protein | YP_277482.1| hypothetical protein JK_42 [Enterobacteria phage JK06] | 6.00E-66 |  |  |
| 11 | 2547..2924 | + | 378 | 14254 | 8.6 | 125 | conserved hypothetical protein | AEI91263.1| gp63 [Escherichia phage phiEB49] | 2.00E-46 |  |  |
| 12 | 2917..3111 | + | 195 | 7309 | 5.2 | 64 | hypothetical protein |  |  |  |  |
| 13 | 3249..3371 | + | 123 | 4811 | 9.2 | 40 | hypothetical protein |  |  |  |  |
| 14 | 3479..3664 | + | 186 | 6805 | 9.4 | 61 | conserved hypothetical protein; putative cytochrome | AEI91261.1| gp61 [Escherichia phage phiEB49] | 9.00E-14 | 1cpq_A Cytochrome C' | 91.4 |
| 15 | 3764..4279 | + | 516 | 19287 | 5.8 | 171 | terminase, small subunit | AEI91260.1| gp60 [Escherichia phage phiEB49] | E-107 |  |  |
| 16 | 4300..5844 | + | 1545 | 58508 | 6.6 | 514 | terminase, large subunit | AEI91258.1| gp58 [Escherichia phage phiEB49]; Pfam PF03237.10 Terminase_6 | 0 |  |  |
| 17 | 5894..7168 | + | 1275 | 46336 | 4.6 | 424 | portal protein | YP_398967.1| putative portal protein [Enterobacteria phage RTP]; Pfam PF06381.6 DUF1073 | 0 |  |  |
| 18 | 7128..7892 | + | 765 | 28440 | 6.0 | 254 | putative phage head morphogenesis protein | YP_277472.1| head morphogenesis protein [Enterobacteria phage JK06] | 0 |  |  |
| 19 | 7882..8991 | + | 1110 | 39761 | 5.0 | 369 | major head subunit | YP_277469.1| hypothetical protein JK_31 [Enterobacteria phage JK06]; Pfam PF09979.4 DUF2213 | 0 |  |  |
| 20 | 9003..9470 | + | 468 | 15635 | 5.4 | 155 | conserved hypothetical protein; structural protein | YP_277467.1| hypothetical protein JK_28 [Enterobacteria phage JK06] | 2.00E-69 | 3gqh_A Preneck appendage protein | 48.9 |
| 21 | 9564..11009 | + | 1446 | 48542 | 4.9 | 481 | conserved hypothetical protein; collagen-like protein | YP_277466.1| hypothetical DNA polymerase I [Enterobacteria phage JK06]; Pfam PF01391.13 Collagen | 9.00E-96 | 3hqv_A Collagen alpha-1(I) chain | 99.7 |
| 22 | 11101..12048 | + | 948 | 34447 | 5.3 | 315 | conserved hypothetical protein | YP_277465.1| hypothetical protein JK_25 [Enterobacteria phage JK06]; Pfam PF09950.4 DUF2184 | 0 |  |  |
| 23 | 12112..12597 | + | 486 | 18488 | 9.4 | 161 | putative HNH endonuclease | YP_398984.1| putative HNH endonuclease [Enterobacteria phage RTP]; Pfam PF13392.1 HNH_3 | 1.00E-50 |  |  |
| 24 | 12626..12871 | + | 246 | 8930 | 5.7 | 81 | conserved hypothetical protein | YP_277464.1| hypothetical protein JK_24 [Enterobacteria phage JK06] | 6.00E-41 |  |  |
| 25 | 12916..13317 | + | 402 | 14900 | 6.6 | 133 | conserved hypothetical protein; head-tail connector protein | AEI91249.1| gp49 [Escherichia phage phiEB49]; Pfam PF13262.1 DUF4054 | 8.00E-70 | 2kbz_A GP15, phage SPP1 gp15 protein | 93.0 |
| 26 | 13314..13682 | + | 369 | 13489 | 8.0 | 122 | conserved hypothetical protein; head-tail joining protein | AEI91248.1| gp48 [Escherichia phage phiEB49] | 4.00E-80 | 1k0h_A Gpfii; twisted beta-sandwich | 96.4 |
| 27 | 13675..14103 | + | 429 | 15710 | 8.0 | 142 | conserved hypothetical protein | AEI91247.1| gp47 [Escherichia phage phiEB49] | 1.00E-96 |  |  |
| 28 | 14093..14491 | + | 399 | 14733 | 5.3 | 132 | conserved hypothetical protein; tail terminator protein | YP_277458.1| hypothetical protein JK_18 [Enterobacteria phage JK06]; Pfam PF13554.1 DUF4128 | 4.00E-94 | 3fz2_A Minor tail protein U | 94.7 |
| 29 | 14506..15156 | + | 651 | 23430 | 4.6 | 216 | major tail protein | YP_277457.1| hypothetical protein JK_17 [Enterobacteria phage JK06]; Pfam PF08813.6 Phage_tail_3 | E-151 |  |  |
| 30 | 15230..15544 | + | 315 | 11840 | 5.0 | 104 | conserved hypothetical protein | YP_277456.1| hypothetical protein JK_16 [Enterobacteria phage JK06]; Pfam PF08748.6 DUF1789 | 1.00E-56 |  |  |
| 31 | 15607..15861 | + | 255 | 10004 | 4.4 | 84 | conserved hypothetical protein | YP_277454.1| hypothetical protein T1P39 [Enterobacteria phage JK06]; Pfam PF08809.6 DUF1799 | 1.00E-55 |  |  |
| 32 | 15896..18949 | + | 3054 | 108762 | 5.3 | 1017 | tail tape measure protein | AEI91241.1| gp41 [Escherichia phage phiEB49]; Pfam PF06791.8 TMP_2 | 0 |  |  |
| 33 | 18981..19331 | + | 351 | 13143 | 5.7 | 116 | minor tail protein | YP_277448.1| putative minor tail protein [Enterobacteria phage JK06]; Pfam PF05939.8 Phage_min_tail | 3.00E-78 |  |  |
| 34 | 19404..19535 | + | 132 | 4970 | 3.8 | 43 | hypothetical protein |  |  |  |  |
| 35 | 19655..20419 | + | 765 | 28168 | 5.3 | 254 | minor tail protein | YP_277447.1| hypothetical protein JK_7 [Enterobacteria phage JK06]; Pfam PF05100.7 Phage_tail_L | 0 |  |  |
| 36 | 20429..21175 | + | 747 | 28667 | 5.1 | 248 | putative minor tail protein | YP_277445.1| hypothetical GP19 [Enterobacteria phage JK06]; Pfam PF00877.14 NLPC_P60 | 0 |  |  |
| 37 | 21156..21722 | + | 567 | 20022 | 9.5 | 188 | putative tail assembly protein | AEI91235.1| gp35 [Escherichia phage phiEB49]; 1 TMD; Pfam PF06805.7 Lambda_tail_I | 7.00E-95 |  |  |
| 38 | 21803..25189 | + | 3387 | 124523 | 4.6 | 1128 | putative tail fiber protein | YP_277442.1| tail fiber [Enterobacteria phage JK06]; Pfam PF13550.1 Phage-tail_3 | 0 |  |  |
| 39 | 25221..26183 | - | 963 | 33735 | 4.9 | 320 | conserved hypothetical protein | YP_277441.1| hypothetical protein JK_1 [Enterobacteria phage JK06] | 0 |  |  |
| 40 | 26183..26428 | - | 246 | 8210 | 8.5 | 81 | conserved hypothetical protein | AEI91232.1| gp32 [Escherichia phage phiEB49] | 2.00E-38 |  |  |
| 41 | 27017..27646 | + | 630 | 24716 | 5.6 | 209 | conserved hypothetical protein; putative transcriptional regulator | YP_277522.1| hypothetical protein JK_82 [Enterobacteria phage JK06]; Pfam PF09669.5 Phage_pRha | E-119 | 2a61_A Transcriptional regulator | 87.0 |
| 42 | 27720..27914 | + | 195 | 7277 | 4.9 | 64 | conserved hypothetical protein | AEI91229.1| gp29 [Escherichia phage phiEB49] | 5.00E-23 |  |  |
| 43 | 27918..28886 | + | 969 | 36446 | 5.8 | 322 | putative exodeoxyribonuclease VIII (RecE) | AEI91228.1| gp28 [Escherichia phage phiEB49]; Pfam PF12684.2 DUF3799 | 0 |  |  |
| 44 | 28943..29590 | + | 648 | 23855 | 5.8 | 215 | putative recombination protein | AEI91227.1| gp27 [Escherichia phage phiEB49]; Pfam PF04404.7 ERF | E-127 |  |  |
| 45 | 29638..30060 | + | 423 | 16385 | 6.7 | 140 | single-stranded DNA binding protein | YP_277517.1| hypothetical protein JK_77 [Enterobacteria phage JK06] | 9.00E-77 |  |  |
| 46 | 30092..32809 | - | 2718 | 97049 | 4.7 | 905 | putative tail fiber protein | YP_277515.1| hypothetical tail fiber [Enterobacteria phage JK06] | 0 |  |  |
| 47 | 32888..33811 | - | 924 | 34495 | 6.3 | 307 | DNA primase | YP_277514.1| hypothetical alpha replication protein [Enterobacteria phage JK06]; Pfam PF08273.7 Prim_Zn_Ribbon | 0 |  |  |
| 48 | 33864..34337 | - | 474 | 18224 | 10.4 | 157 | putative transcriptional regulator | YP_277513.1| hypothetical transcriptional regulator [Enterobacteria phage JK06] | E-101 | 1zug_A Phage 434 CRO protein | 99.6 |
| 49 | 34430..36373 | + | 1944 | 72383 | 8.5 | 647 | ATP-dependent helicase | AEI91221.1| gp21 [Escherichia phage phiEB49]; Pfam PF00271.26 Helicase_C | 0 |  |  |
| 50 | 36373..36762 | + | 390 | 14349 | 8.5 | 129 | conserved hypothetical protein; putative Holliday-junction resolvase | AEI91220.1| gp20 [Escherichia phage phiEB49]; Pfam PF08774.6 VRR_NUC | 7.00E-87 | 1ob8_A Holliday-junction resolvase | 96.8 |
| 51 | 36821..37015 | + | 195 | 7406 | 4.2 | 64 | conserved hypothetical protein | YP_277508.1| hypothetical protein JK_68 [Enterobacteria phage JK06] | 9.00E-40 | 3p6j_A De novo designed beta-trefoil architecture | 89.2 |
| 52 | 37015..37233 | + | 219 | 8742 | 4.1 | 72 | conserved hypothetical protein | AEI91218.1| gp18 [Escherichia phage phiEB49] | 1.00E-36 |  |  |
| 53 | 37230..37430 | + | 201 | 7546 | 4.3 | 66 | conserved hypothetical protein | YP_399001.1| hypothetical protein rtp57 [Enterobacteria phage RTP] | 3.00E-14 |  |  |
| 54 | 37505..37630 | + | 126 | 4788 | 5.0 | 41 | conserved hypothetical protein | YP_399002.1| hypothetical protein rtp58 [Enterobacteria phage RTP] | 9.00E-21 |  |  |
| 55 | 37627..37866 | + | 240 | 9003 | 9.6 | 79 | conserved hypothetical protein | YP_277505.1| hypothetical protein JK_65 [Enterobacteria phage JK06] | 3.00E-44 |  |  |
| 56 | 37863..38120 | + | 258 | 10001 | 4.3 | 85 | conserved hypothetical protein | AEI91214.1| gp14 [Escherichia phage phiEB49] | 9.00E-56 |  |  |
| 57 | 38272..39405 | + | 1134 | 42186 | 6.3 | 377 | conserved hypothetical protein; putative exonuclease | YP_399005.1| hypothetical protein rtp61 [Enterobacteria phage RTP] | 0 | 1su1_A Hypothetical protein YFCE | 99.9 |
| 58 | 39471..39668 | + | 198 | 7458 | 4.7 | 65 | hypothetical protein |  |  |  |  |
| 59 | 39745..39930 | + | 186 | 6669 | 5.0 | 61 | conserved hypothetical protein | AEI91211.1| gp11 [Escherichia phage phiEB49] | 4.00E-32 |  |  |
| 60 | 39930..40118 | + | 189 | 6865 | 7.6 | 62 | hypothetical protein |  |  |  |  |
| 61 | 40131..40304 | + | 174 | 6445 | 4.2 | 57 | conserved hypothetical protein | AEI91210.1| gp10 [Escherichia phage phiEB49] | 6.00E-35 | 1yez_A MM1357; MAR30, autostructure | 88.2 |
| 62 | 40419..40634 | + | 216 | 7637 | 9.0 | 71 | putative holin | AEI91209.1| gp9 [Escherichia phage phiEB49]; 1 TMD | 5.00E-32 |  |  |
| 63 | 40634..41116 | + | 483 | 17257 | 9.5 | 160 | lysin (lysozyme) | YP_277498.1| hypothetical phage-related lysozyme [Enterobacteria phage JK06]; Pfam PF00959.14 Phage_lysozyme | E-106 |  |  |
| 64 | 41098..41502 | + | 405 | 13937 | 5.7 | 134 | Rz1A protein | AEI91207.1| gp7 [Escherichia phage phiEB49]; 1-2 TMD | 4.00E-82 |  |  |
| 65 | 41516..41851 | - | 336 | 12872 | 8.3 | 111 | conserved hypothetical protein; putative transcriptional regulator | YP_277496.1| YdbL [Enterobacteria phage JK06] | 1.00E-77 | 3oou_A LIN2118 protein | 85.7 |
| 66 | 41856..43439 | - | 1584 | 58029 | 6.1 | 527 | conserved hypothetical protein; putative replication protein | AEI91205.1| gp5 [Escherichia phage phiEB49]; Pfam PF13148.1 DUF3987 | 0 | 1w5s_A Origin recognition complex subunit 2 ORC2 | 95.9 |
| 67 | 43582..43794 | + | 213 | 8490 | 9.3 | 70 | conserved hypothetical protein | YP_277491.1| hypothetical protein JK_51 [Enterobacteria phage JK06] | 5.00E-40 |  |  |
| 68 | 43940..44149 | - | 210 | 7706 | 7.6 | 69 | conserved hypothetical protein | YP_004327403.1| conserved uncharacterised protein [Salmonella phage Vi01] | 5.00E-10 |  |  |
| 69 | 44146..44499 | - | 354 | 13414 | 9.6 | 117 | conserved hypothetical protein | YP_277490.1| hypothetical protein JK_50 [Enterobacteria phage JK06] | 5.00E-58 |  |  |
| 70 | 44525..44827 | - | 303 | 11311 | 5.3 | 100 | hypothetical protein |  |  |  |  |
| 71 | 44902..45054 | - | 153 | 5700 | 7.9 | 50 | conserved hypothetical protein | AEI91203.1| gp3 [Escherichia phage phiEB49] | 1.00E-22 |  |  |
| 72 | 45055..45294 | - | 240 | 8810 | 8.9 | 79 | conserved hypothetical protein | AEI91202.1| gp2 [Escherichia phage phiEB49] | 1.00E-49 |  |  |
| 73 | 45305..45484 | - | 180 | 6634 | 4.4 | 59 | conserved hypothetical protein | YP_399018.1| hypothetical protein rtp74 [Enterobacteria phage RTP] | 3.00E-29 |  |  |
| 74 | 45474..45668 | - | 195 | 7548 | 4.4 | 64 | hypothetical protein |  |  |  |  |
|  |  |  |  |  |  |  | BLAST and motifs searches conducted December 2, 2011 | |  |  |  |
|  |  |  |  |  |  |  | TMD = transmembrane domain |  |  |  |  |
