# Supplementary material for: Endemic bacteriophages: a cautionary tale for evaluation of bacteriophage therapy and other interventions for infection control in animals
Source: Virol J. 2012 Sep 17;9:207. doi: 10.1186/1743-422X-9-207 (PMC3496638; doi:10.1186/1743-422X-9-207)
Supplement: Additional file 2 — Figure S1. Mass spectral analysis of three Rogue1 structural proteins. [file 1743-422X-9-207-S2.pptx]

## Slide 1
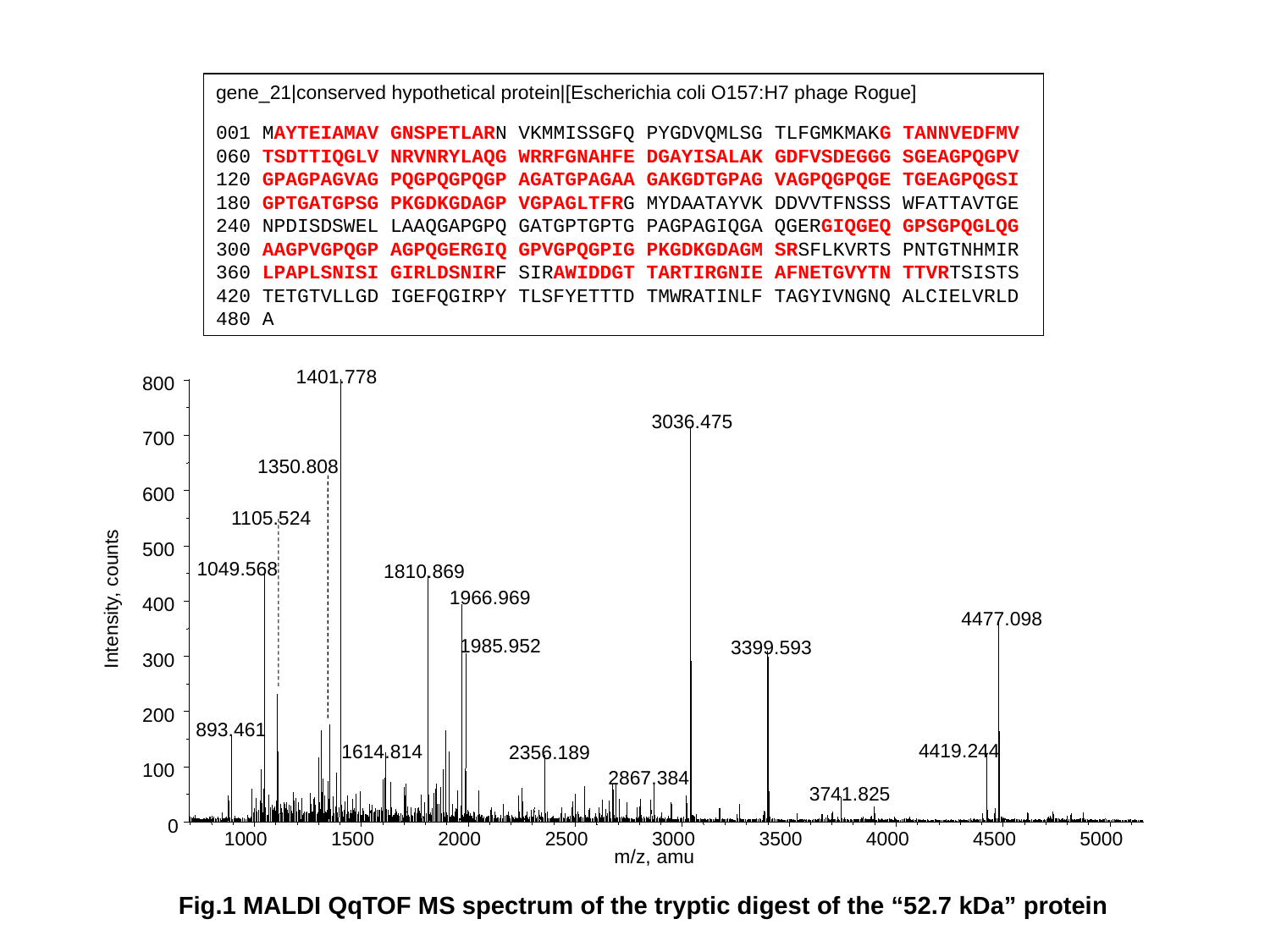

gene_21|conserved hypothetical protein|[Escherichia coli O157:H7 phage Rogue]
001 MAYTEIAMAV GNSPETLARN VKMMISSGFQ PYGDVQMLSG TLFGMKMAKG TANNVEDFMV
060 TSDTTIQGLV NRVNRYLAQG WRRFGNAHFE DGAYISALAK GDFVSDEGGG SGEAGPQGPV
120 GPAGPAGVAG PQGPQGPQGP AGATGPAGAA GAKGDTGPAG VAGPQGPQGE TGEAGPQGSI
180 GPTGATGPSG PKGDKGDAGP VGPAGLTFRG MYDAATAYVK DDVVTFNSSS WFATTAVTGE
240 NPDISDSWEL LAAQGAPGPQ GATGPTGPTG PAGPAGIQGA QGERGIQGEQ GPSGPQGLQG
300 AAGPVGPQGP AGPQGERGIQ GPVGPQGPIG PKGDKGDAGM SRSFLKVRTS PNTGTNHMIR
360 LPAPLSNISI GIRLDSNIRF SIRAWIDDGT TARTIRGNIE AFNETGVYTN TTVRTSISTS
420 TETGTVLLGD IGEFQGIRPY TLSFYETTTD TMWRATINLF TAGYIVNGNQ ALCIELVRLD
480 A
1401.778
800
3036.475
700
1350.808
600
1105.524
500
1049.568
1810.869
1966.969
Intensity, counts
400
4477.098
1985.952
3399.593
300
200
893.461
4419.244
1614.814
2356.189
100
2867.384
3741.825
0
1000
1500
2000
2500
3000
3500
4000
4500
5000
m/z, amu
Fig.1 MALDI QqTOF MS spectrum of the tryptic digest of the “52.7 kDa” protein

## Slide 2
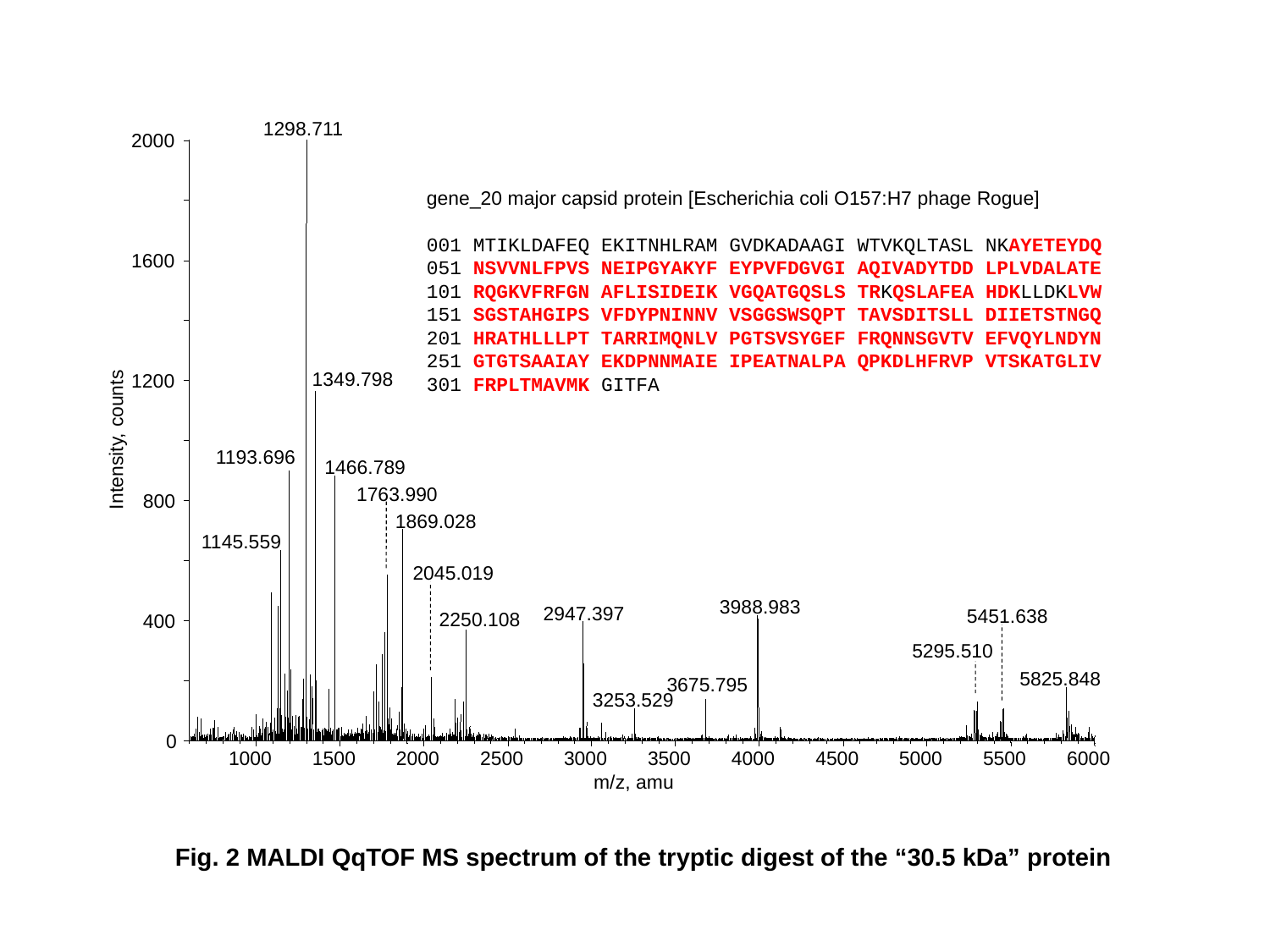

1298.711
2000
gene_20 major capsid protein [Escherichia coli O157:H7 phage Rogue]
001 MTIKLDAFEQ EKITNHLRAM GVDKADAAGI WTVKQLTASL NKAYETEYDQ
051 NSVVNLFPVS NEIPGYAKYF EYPVFDGVGI AQIVADYTDD LPLVDALATE
101 RQGKVFRFGN AFLISIDEIK VGQATGQSLS TRKQSLAFEA HDKLLDKLVW
151 SGSTAHGIPS VFDYPNINNV VSGGSWSQPT TAVSDITSLL DIIETSTNGQ
201 HRATHLLLPT TARRIMQNLV PGTSVSYGEF FRQNNSGVTV EFVQYLNDYN
251 GTGTSAAIAY EKDPNNMAIE IPEATNALPA QPKDLHFRVP VTSKATGLIV
301 FRPLTMAVMK GITFA
1600
1349.798
1200
Intensity, counts
1193.696
1466.789
1763.990
800
1869.028
1145.559
2045.019
3988.983
2947.397
5451.638
2250.108
400
5295.510
5825.848
3675.795
3253.529
0
1000
1500
2000
2500
3000
3500
4000
4500
5000
5500
6000
m/z, amu
Fig. 2 MALDI QqTOF MS spectrum of the tryptic digest of the “30.5 kDa” protein

## Slide 3
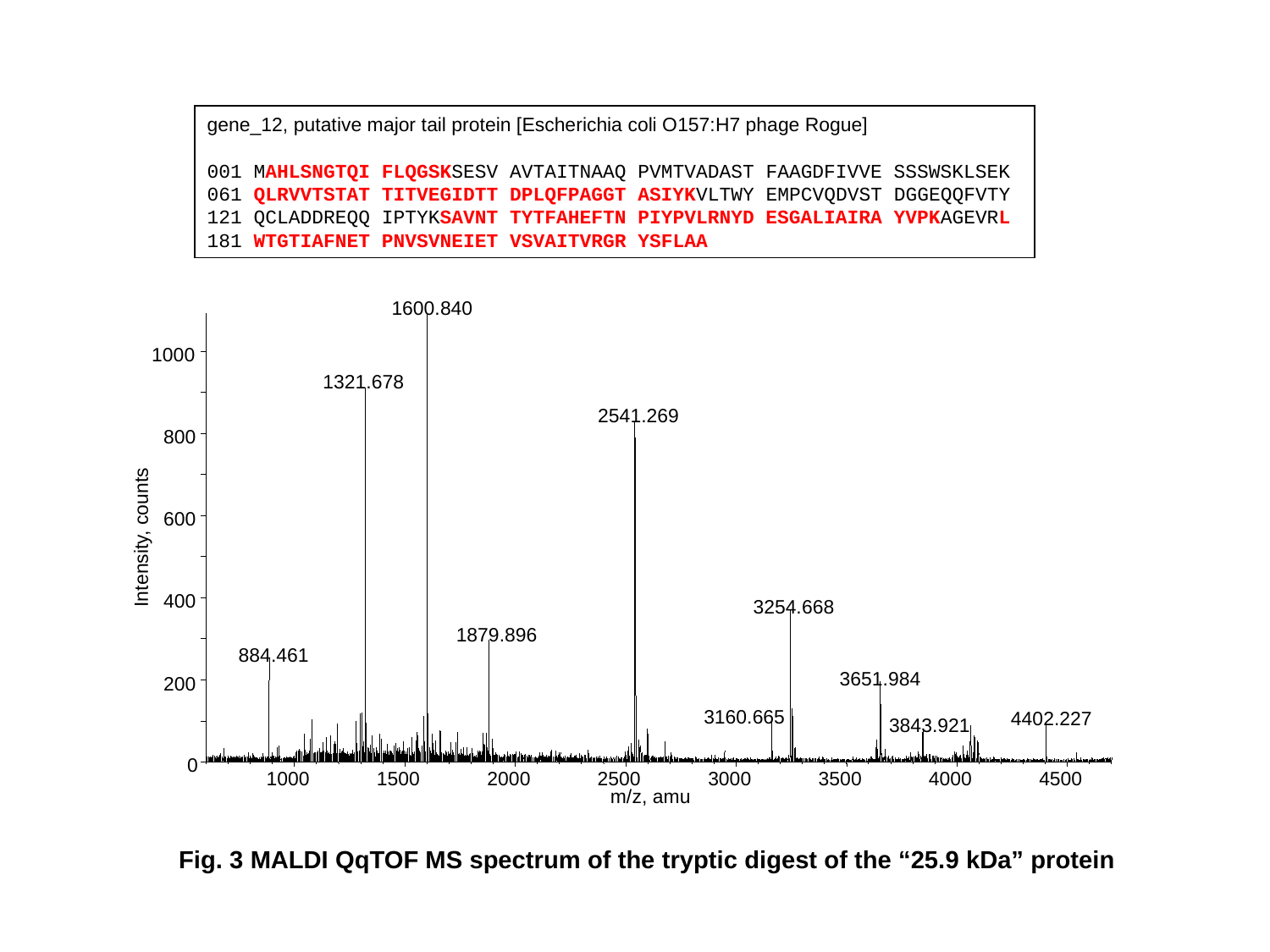

gene_12, putative major tail protein [Escherichia coli O157:H7 phage Rogue]
001 MAHLSNGTQI FLQGSKSESV AVTAITNAAQ PVMTVADAST FAAGDFIVVE SSSWSKLSEK
061 QLRVVTSTAT TITVEGIDTT DPLQFPAGGT ASIYKVLTWY EMPCVQDVST DGGEQQFVTY
121 QCLADDREQQ IPTYKSAVNT TYTFAHEFTN PIYPVLRNYD ESGALIAIRA YVPKAGEVRL
181 WTGTIAFNET PNVSVNEIET VSVAITVRGR YSFLAA
1600.840
1000
1321.678
2541.269
800
600
Intensity, counts
400
3254.668
1879.896
884.461
3651.984
200
3160.665
4402.227
3843.921
0
1000
1500
2000
2500
3000
3500
4000
4500
m/z, amu
Fig. 3 MALDI QqTOF MS spectrum of the tryptic digest of the “25.9 kDa” protein
