# Supplementary material for: Endemic bacteriophages: a cautionary tale for evaluation of bacteriophage therapy and other interventions for infection control in animals
Source: Virol J. 2012 Sep 17;9:207. doi: 10.1186/1743-422X-9-207 (PMC3496638; doi:10.1186/1743-422X-9-207)
Supplement: Additional file 3 — Table S2. Protein sequence identification of Rogue1 phage by MS/MS measurements of the peptides and database search. [file 1743-422X-9-207-S3.doc]

**Additional file 3: Table S2.** Protein sequence identification of Rogue1 phage by MS/MS measurements of the peptides and database search

| **m/z**  **(Meas.)** | **[MH+]**  **(calc.)** | **Error**  **(Da)** | **Peptide** | **Sequence** |
| --- | --- | --- | --- | --- |
| 52.7kDa band: >gene_21|conserved hypothetical protein|[Escherichia coli O157:H7 phage Rogue]; MW: 48541 Da; Sequence coverage 59.5% | | | | |
| 893.461 | 893.463 | -0.002 | 76-82 | (R)YLAQGWR(R) |
| 1049.568 | 1049.564 | 0.004 | 76-83 | (R)YLAQGWRR(F) |
| 1105.524 | 1105.527 | -0.003 | 384-393 | (R)AWIDDGTTAR (T) |
| 1350.808 | 1350.810 | -0.002 | 361-373 | (R)LPAPLSNISIGIR (L) |
| 1401.778 | 1401.785 | -0.007 | 318-332 | (R)GIQGPVGPQGPIGPK (G) |
| 1614.814 | 1614.823 | -0.009 | 193-209 | (K)GDKGDAGPVGPAGLTFR(G) |
| 1810.869 | 1810.876 | -0.007 | 84-100 | (R)FGNAHFEDGAYISALAK (G) |
| 1966.969 | 1966.977 | -0.008 | 83-100 | (R)RFGNAHFEDGAYISALAK(G) |
| 1983.938 | 1983.937 | 0.001 | 2-19 | (-)AYTEIAMAVGNSPETLAR(N) |
| 1985.952 | 1985.956 | -0.004 | 397-414 | (R)GNIEAFNETGVYTNTTVR (T) |
| 2356.189 | 2356.189 | 0.000 | 394-414 | (R)TIRGNIEAFNETGVYTNTTVR (T) |
| 3036.475 | 3036.488 | -0.013 | 285-317 | (R)GIQGEQGPSGPQGLQGAAGPVGPQGPAGPQGER(G) |
| 3399.593 | 3399.605 | -0.006 | 154-192 | (R)GDTGPAGVAGPQGPQGETGEAGPQGSIGPTGATGPSGPK(G) |
| 4419.244 | 4419.255 | -0.011 | 285-332 | (R)GIQGEQGPSGPQGLQGAAGPVGPQGPAGPQGERGIQGPVG PQGPIGPK (G) |
| 4477.098 | 4477.104 | -0.016 | 101-153 | (K)GDFVSDEGGGSGEAGPQGPVGPAGPAGVAGPQGPQGPQGPAGATGPAGAAGAK(G) |
|  |  |  |  |  |
| 30.5 kDa band: >gene_20 |major capsid protein| [Escherichia coli O157:H7 phage Rogue]; MW: 34446 Da; Sequence coverage: 83.5% | | | | |
| 1145.559 | 1145.559 | 0.000 | 134-143 | (K)QSLAFEAHDK(L) |
| 1193.696 | 1193.700 | -0.004 | 203-213 | (R)ATHLLLPTTAR(R) |
| 1298.711 | 1298.721 | -0.010 | 284-294 | (K)DLHFRVPVTSK(A) |
| 1349.798 | 1349.801 | -0.003 | 203-214 | (R)ATHLLLPTTARR(I) |
| 1466.789 | 1466.789 | 0.000 | 108-120 | (R)FGNAFLISIDEIK(V) |
| 1763.990 | 1763.991 | -0.001 | 295-310 | (K)ATGLIVFRPLTMAVMK(G) (Met-OX) |
| 1869.028 | 1869.027 | 0.001 | 105-120 | (K)VFRFGNAFLISIDEIK(V) |
| 2045.019 | 2045.016 | 0.003 | 215-232 | (R)IMQNLVPGTSVSYGEFFR(Q) |
| 2250.108 | 2250.107 | 0.001 | 263-283 | (K)DPNNMAIEIPEATNALPAQPK(D) (Met-OX) |
| 2947.391 | 2947.400 | -0.009 | 43-68 | (K)AYETEYDQNSVVNLFPVSNEIPGYAK(Y) |
| 3675.795 | 3675.810 | -0.015 | 69-101 | (K)YFEYPVFDGVGIAQIVADYTDDLPLVDALATER(Q) |
| 3988.983 | 3988.985 | -0.002 | 69-104 | (K)YFEYPVFDGVGIAQIVADYTDDLPLVDALATERQGK(V) |
|  |  |  |  |  |
| **25.9 kDa band:** >gene_12 |putative major tail protein|[Escherichia coli O157:H7 phage Rogue]; MW: 23429 Da; Sequence coverage: 53.7% | | | | |
| 884.461 | 884.462 | -0.001 | 209-216 | (R)GRYSFLAA(-) |
| 1321.678 | 1321.675 | 0.003 | 158-169 | (R)NYDESGALIAIR(A) |
| 1600.840 | 1600.844 | -0.004 | 2-16 | (-)AHLSNGTQIFLQGSK(S) |
| 1879.896 | 1879.991 | -0.005 | 158-174 | (R)NYDESGALIAIRAYVPK(A) |
| 2541.269 | 2541.277 | -0.008 | 136-157 | (K)SAVNTTYTFAHEFTNPIYPVLR(N) |
| 3254.668 | 3254.668 | 0.000 | 64-95 | (R)VVTSTATTITVEGIDTTDPLQFPAGGTASIYK(V) |
| 3651.984 | 3651.962 | 0.012 | 61-95 | (K)QLRVVTSTATTITVEGIDTTDPLQFPAGGTASIYK(V) |
| 3843.921 | 3843.934 | -0.013 | 136-169 | (K)SAVNTTYTFAHEFTNPIYPVLRNYDESGALIAIR(A) |
