# Supplementary material for: Endemic bacteriophages: a cautionary tale for evaluation of bacteriophage therapy and other interventions for infection control in animals
Source: Virol J. 2012 Sep 17;9:207. doi: 10.1186/1743-422X-9-207 (PMC3496638; doi:10.1186/1743-422X-9-207)
Supplement: Additional file 4 — Figure S2. ClustalW alignment of Rogue1 gp21 with its homolog phage JK06 protein YP_277466.1. The pentameric repeats are indicated in bold and green. [file 1743-422X-9-207-S4.doc]

**Additional file 4: Figure S2.** ClustalW alignment of Rogue1 gp*21* with its homolog phage JK06 protein

YP_277466.1. The pentameric repeats are indicated in bold and green.

Phage_vB_EcoS_Rogue1 MAYTEIAMAVGNSPETLARNVKMMISSGFQPYGDVQMLSGTLFGMKMAKG 50

Enterobacteria_phage_JK06 MAYTEIAMAVGNSPETLARNVKMMISSGFQPYGDVQMLSGTLFGMKMAKG 50

**************************************************

Phage_vB_EcoS_Rogue1 TANNVEDFMVTSDTTIQGLVNRVNRYLAQGWRRFGNAHFEDGAYISALAK 100

Enterobacteria_phage_JK06 TANNVEDFMVTSDTTIQGLVNRVNRYLAQGWRRFGNAHFEDGAYISALAK 100

**************************************************

Phage_vB_EcoS_Rogue1 GDFVSDEGGGSGEAGPQ**GPVGP**AGPAGVAGPQGPQGPQGPAGATGPAGAA 150

Enterobacteria_phage_JK06 GDFVSDEGGGSGEAGPQG-------------------------------- 118

******************

Phage_vB_EcoS_Rogue1 GAKGDTGPAGVAGPQGPQGETGEAGPQGSIGPTGATGPSGPKGDKGDA**GP** 200

Enterobacteria_phage_JK06 --------------------------------------------------

Phage_vB_EcoS_Rogue1 **VGP**AGLTFRGMYDAATAYVKDDVVTFNSSSWFATTAVTGENPDISDSWEL 250

Enterobacteria_phage_JK06 --------------------------------------------------

Phage_vB_EcoS_Rogue1 LAAQGAPGPQGATGPTGPTGPAGPAGIQGAQGERGIQGEQGPSGPQGLQG 300

Enterobacteria_phage_JK06 --------------------------------------------------

Phage_vB_EcoS_Rogue1 AA**GPVGP**QGPAGPQGERGIQ**GPVGP**QGPIGPKGDKGDAGMSRSFLKVRTS 350

Enterobacteria_phage_JK06 ---------------------PVGPQGPIGPKGDKGDAGMSRSFLKVRTS 147

*****************************

Phage_vB_EcoS_Rogue1 PNTGTNHMIRLPAPLSNISIGIRLDSNIRFSIRAWIDDGTTARTIRGNIE 400

Enterobacteria_phage_JK06 PNTGTNHMIRLPAPLSNISIGIRLDSNIRFSIRAWIDDGTTARTIRGNIE 197

**************************************************

Phage_vB_EcoS_Rogue1 AFNETGVYTNTTVRTSISTSTETGTVLLGDIGEFQGIRPYTLSFYETTTD 450

Enterobacteria_phage_JK06 AFNETGVYTNTTVRTSISTSTETGTVLLGDIGEFQGIRPYTLSFYETTTD 247

**************************************************

Phage_vB_EcoS_Rogue1 TMWRATINLFTAGYIVNGNQALCIELVRLDA 481

Enterobacteria_phage_JK06 TMWRATINLFTAGYIVNGNQALCIELVRLDA 278

*******************************
