# Supplementary figures and images for: Endemic bacteriophages: a cautionary tale for evaluation of bacteriophage therapy and other interventions for infection control in animals
Source: Virol J. 2012 Sep 17;9:207. doi: 10.1186/1743-422X-9-207 (PMC3496638; doi:10.1186/1743-422X-9-207)

## Slide 1
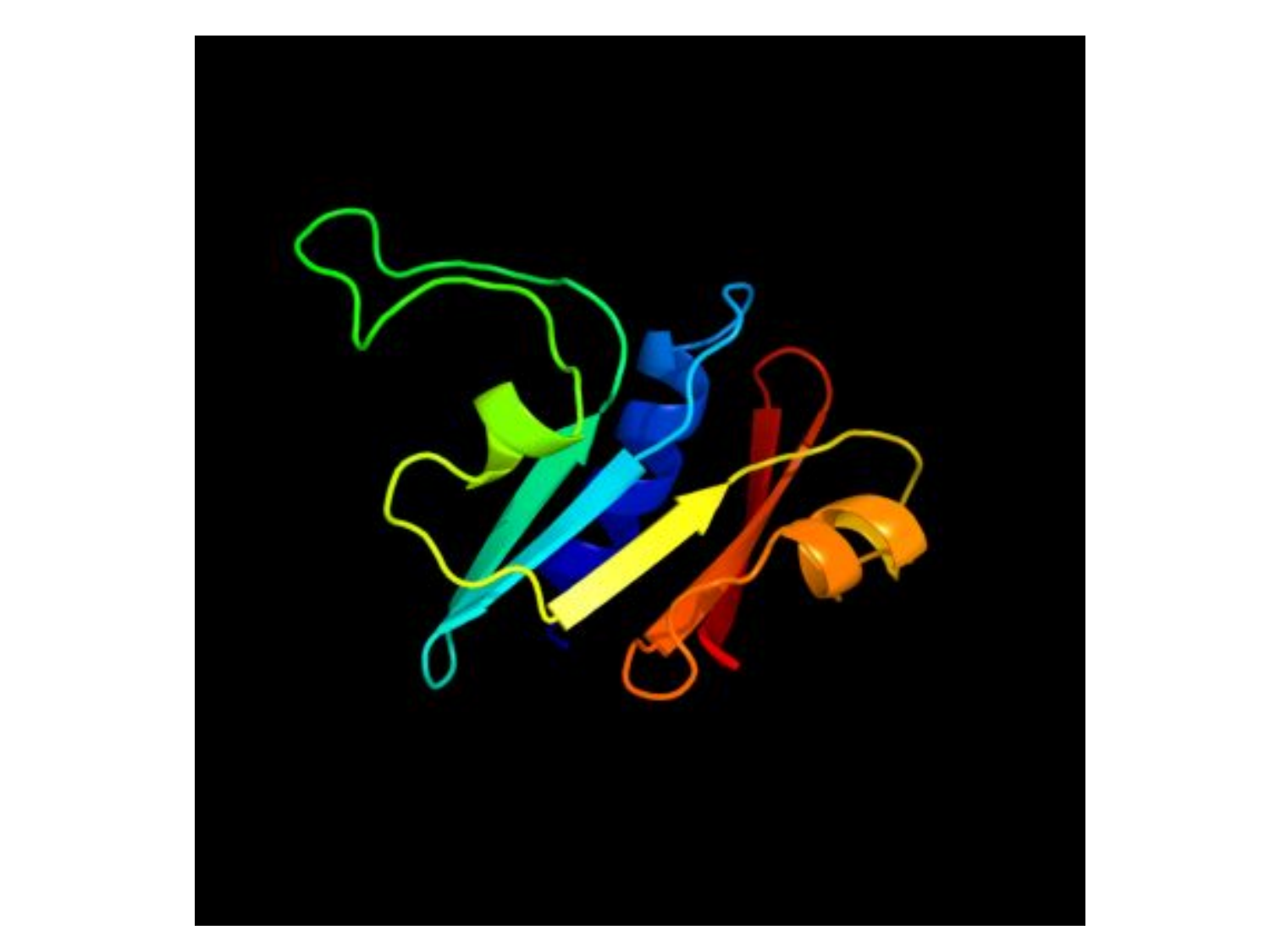

Supplement: Additional file 5 — Figure S3. 3D structure of a portion of gp36 based upon template c2fg0B, determined using Phyre2[39]. [file 1743-422X-9-207-S5.ppt]
